# N-Terminal Helix-Cap in α-Helix 2 Modulates β-State Misfolding in Rabbit and Hamster Prion Proteins

**DOI:** 10.1371/journal.pone.0063047

**Published:** 2013-05-10

**Authors:** Braden Sweeting, Eric Brown, M. Qasim Khan, Avijit Chakrabartty, Emil F. Pai

**Affiliations:** 1 Department of Medical Biophysics, University of Toronto, Toronto, Ontario, Canada; 2 The Campbell Family Cancer Research Institute, Ontario Cancer Institute, Toronto, Ontario, Canada; 3 Department of Biochemistry, University of Toronto, Toronto, Ontario, Canada; 4 Department of Molecular Genetics, University of Toronto, Toronto, Ontario, Canada; National Institute for Agricultural Research, France

## Abstract

Susceptibility of a particular species to prion disease is affected by small differences in the sequence of PrP and correlates with the propensity of its PrP to assume the β-state. A helix-cap motif in the β2−α2-loop of native α-helical rabbit PrP, a resistant species, contains sequence differences that influence intra- and interspecies transmission. To determine the effect the helix-cap motif on β-state refolding propensity, we mutated S170N, S174N, and S170N/S174N of the rabbit PrP helix-cap to resemble that of hamster PrP and conversely, N170S, N174S, and N170S/N174S of hamster PrP to resemble the helix-cap of rabbit PrP. High-resolution crystal structures (1.45–1.6 Å) revealed that these mutations ablate hydrogen-bonding interactions within the helix-cap motif in rabbit PrP^C^. They also alter the β-state-misfolding propensity of PrP; the serine mutations in hamster PrP decrease the propensity up to 35%, whereas the asparagine mutations in rabbit PrP increase it up to 42%. Rapid dilution of rabbit and hamster into β-state buffer conditions causes quick conversion to β-state monomers. Kinetic monitoring using size-exclusion chromatography showed that the monomer population decreases exponentially mirrored by an increase in an octameric species. The monomer-octamer transition rates are faster for hamster than for rabbit PrP. The N170S/N174S mutant of hamster PrP has a smaller octamer component at the endpoint compared to the wild-type, whereas the kinetics of octamer formation in mutant and wild-type rabbit PrP are comparable. These findings demonstrate that the sequence of the β2−α2 helix-cap affects refolding to the β-state and subsequently, may influence susceptibility to prion disease.

## Introduction

Pathogenesis in prion disease involves refolding of the host protein, PrP, from the monomeric, primarily α-helical cellular form (PrP^C^) to a β-sheet enriched, aggregated infectious form (PrP^Sc^) [1]. Susceptibility to prion disease varies depending on the donor species and strain of infectious prion as well as the species and genotype of the recipient. However, some species appear to be susceptible to prions from multiple sources (e.g. bank voles and hamsters [2]–[5]), whereas others show a lower susceptibility to prion disease (e.g. rabbits, dogs, and horses) [6], [7] or are even completely unaffected (birds) [8]. The determining factor in interspecies prion transmission appears to be intrinsic to the amino acid sequence of PrP [9]. Even single amino acid differences between donor and recipient can give rise to a species barrier [10] or confer resistance to conversion of the recipient PrP^C^ to the infectious form PrP^Sc^ [11].

**Figure 1:**
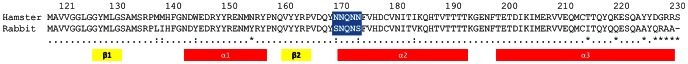
Alignment of Hamster and Rabbit PrP 121–231 Amino Acid Sequences. The β2−α2 helix cap is highlighted in blue and the secondary structure locations are shown below.

The sequence of PrP is highly conserved among mammals. Mutations in PrP could affect susceptibility and transmission of prion disease by causing changes in the structure of PrP^C^ and/or the mechanism of its conversion to PrP^Sc^. The structure of PrP^C^ from many different species has been determined by X-ray crystallography and NMR spectroscopy [12]–[17], revealing that it is highly conserved between species and the differences in amino acid sequence have little effect on the overall fold. The loop between the second β-strand and the second α-helix (the β2−α2 loop) is one region of sequence diversity among species (Fig. 1) and has been often cited as a region of interest in the conversion of PrP^C^ to PrP^Sc^ as well as in its interactions with other proteins [18]. NMR measurements have shown that single amino acid changes in this loop can cause differential backbone mobility. Of more functional importance, they can also lead to the spontaneous onset of disease when expressed in an *in vivo* model [16], [19]. These latter observations suggest that interactions within the β2−α2 loop may play a key role in the conversion to the infectious form.

The investigation of the effect of PrP sequence differences on the mechanism of PrP conversion has been severely hampered by the great difficulty of obtaining samples of purified infectious PrP^Sc^ suitable for high resolution structural studies. Using recombinant PrP, several groups have been able to generate PrP refolded into a β-sheet enriched, oligomeric state under low pH and mild denaturing conditions (β-oligomer or β-state) [20]–[24]. For some of those constructs, toxicity and infectivity could be established [25], [26]. Recent work in our laboratories has shown that the propensity of PrP to form the β-state correlates with the susceptibility of that species to prion disease. Additionally we found that rabbits, a species with low susceptibility to prion disease possess PrP with a helix-capping motif in the β2−α2 loop, which appears to hamper the formation of the β-state.

In this paper we now demonstrate that the presence of either serine (rabbit) or asparagine (hamster) residues in positions 170 and 174 of PrP not only affect the secondary structure of the β2−α2 loop but also the propensity with which the prion protein misfolds into β-state-rich octamers.

## Materials and Methods

### Molecular Biology

The construction of the expression vector has been published [27]. Site-directed mutagenesis was performed using QuikChange mutagenesis kits (Stratagene, La Jolla, CA, USA) as per manufacturer’s instructions.

### Protein Expression and Purification

All wild-type and mutant constructs of hamster and rabbit PrP 121–231 were expressed using a pET28a vector (Novagen, Gibbstown, NJ, USA). Proteins were expressed as inclusion bodies in the *E. coli* BL21 AI strain (Invitrogen, Carlsbad, CA, USA). PrP^C^ was then refolded and purified using a method adopted from Zahn et al. [28].

### Crystallization

Purified mutant rabbit proteins were crystallized using microseeding techniques. Seeds were produced from crystals of wild-type rabbit PrP^C^ grown as described in Kahn et al. [27]. The resultant crystals of the mutant rabbit proteins were crushed, diluted 10^4^–10^6^ fold and used for an additional round of microseeding. Crystals were then grown using the hanging drop vapour diffusion method in a solution of sodium cacodylate pH 6.5 with 2.0–3.0 M sodium chloride as precipitant. The crystals grew as large flat plates in space group P2_1_2_1_2_1_ and their unit cell axes did not differ by more than 0.1 Å from a = 29.6 Å, b = 86.2 Å, and c = 87.1 Å. The crystals were flash-frozen at 100 K using 30% (v/v) glycerol as cryoprotectant.

### Crystal Structure Determination

Each diffraction data set was collected from a single crystal at beamline 08ID-1 at the Canadian Macromolecular Crystallography Facility (Canadian Light Source, Saskatoon, SK, Canada). The diffraction data were processed using XDS [29] and the phase problem was solved with the help of molecular replacement techniques using Phaser [30] and employing the wild-type rabbit PrP^C^ structure (PDB ID: 3O79) as search model. The structure was refined using a combination of the program packages RefMac [31], PHENIX [32] and Coot [33]. Statistics for data collection and refinement are given in Table 1. Coordinates and structure factors have been deposited in the Research Collaboratory for Structural Bioinformatics PDB under accession codes 4 HMR, 4 HMM and 4 HLS.

**Table 1:** Data collection and refinement statistics for the crystal structures of S170N, S174N and S170N/S174N mutants of rabbit PrP^C^ 121–230

Data Statistics	S174N	S170N	DBL
Space group	P2_1_2_1_2_1_	P2_1_2_1_2_1_	P2_1_2_1_2_1_
Cell constants			
a (Å)	29.6	29.5	29.5
b (Å)	86.3	86.1	86.4
c (Å)	87.1	87.0	87.1
Resolution (Å)	30–1.5 (1.60–1.50)	60–1.45 (1.50–1.45)	60–1.60 (1.70–1.60)
Overall reflections	174,057 (30,353)	300,690 (8,742)	216 428 (35,877)
Unique reflections	36,582 (6,321)	39,174 (2,798)	30,243 (4,938)
Redundancy	4.8 (4.8)	7.7 (3.1)	7.2 (7.3)
Completeness (%)	99.8 (99.9)	97.3 (73.2)	99.7 (99.4)
R merge^b^	11.6 (46.0)	5.9 (53.8)	7.9 (37.2)
<*I*>/σ*I*	17.6 (4.6)	24.3 (3.6)	23.0 (5.3)
**Refinement Statistics**			
Final *R*_cryst_ (%)	14.7	14.6	15.5
*R*_free_ (%)	19.3	18.0	20.7
Solvent (%)	40.1	39.9	40.1
No. of protein molecules	2	2	2
No. of all atoms	1888	1954	1892
No. of water molecules	148	186	126
No. of sodium ions	3	6	2
No. of chloride ions	2	2	3
Average B-factor (Å^2^)	15.8	18.2	20.8
Ramachandran plot			
Most favorable	99.5%	99.5%	98.9%
R.M.S.D. from ideal geometry			
Lengths (Å)	0.016	0.016	0.013
Angles (°)	1.532	1.731	1.505

a Values in parenthesis are for the outer shell.

b *R* = ∑_hkl_∑_i_|I_i_(*hkl*) – [I(*hkl*)]|//∑*hkl*∑_i_ *I*(*hkl*), where *I*(*hkl*) is the intensity of reflection *hkl*, ∑*hkl* is the sum over all reflections and ∑_i_ is the sum over *i* measurements of reflection *hkl*.

c *R* = ∑*hkl*||F_obs_| – |F_calc_||/∑_hkl_|F_obs_|, where *F*_obs_ and *F*_calc_ are the observed and calculated structure-factor amplitutes, respectively.

*R*_free_ is calculated for a randomly chosen 5% of reflections that were not used for structure refinement and *R*_work_ is calculated for the remaining reflections.

### Unfolding Curves

Purified PrP^C^ protein stocks of 100 µM were dialyzed into 50 mM sodium phosphate pH 7.0, 80 mM NaCl, 0.5 mM EDTA for urea denaturation experiments at pH 7.0. PrP stocks were dialyzed in 50 mM sodium acetate pH 5.0 or pH 4.5, 80 mM, 0.5 mM EDTA for β-state propensity measurements. Stocks were then diluted 10-fold to a final concentration of 10 µM into increasing concentrations of urea from 0–9 M in buffer solutions identical to their respective dialysis buffers, followed by incubation at room temperature for a minimum of 3 days to allow them to reach equilibrium.

Circular dichroism ellipticity was then measured using a 1 mm path length in a quartz cuvette on a Jasco J-815 CD Spectrometer (Easton, MD, USA). Ellipticity values were measured at 220 nm and 229 nm every 0.5 s for 120 s and averaged. The free energy of unfolding (**ΔG_unfolding_**) of wild-type and mutant PrP at pH 7 were computed by fitting the data to a 2–state (N→U) transition model. The proportion of β-state was determined using the method described by Khan et al. [27].

### Time-resolved Size Exclusion Chromatography and Circular Dichroism

Concentrated stocks (100 µM) of wild-type rabbit and hamster PrP^C^ and the double mutants S170N/S174N rabbit PrP^C^ and N170S/N174S hamster PrP^C^ were dialyzed into 50 mM sodium acetate pH 4.0, 80 mM NaCl, 0.5 mM EDTA. At *t* = 0, the concentrated protein stock was diluted to 10 µM and a final concentration of 4 M urea. A 200 µL sample was immediately injected onto a Superdex S200 10/30 column equilibrated in identical buffer and run at 0.5 ml/min while monitored using the absorbance at 280 nm. Additional samples of 200 µL were injected at indicated time intervals. At t = 0 and t = 4 hrs, CD wavelength scans were performed on the urea diluted samples using a 1 mm path length in a quartz cuvette between 205–250 nm.

## Results

### Crystal Structures of S174N, S170N and S170N/S174N Mutants of Rabbit PrP^C^ 121–230

PrP from hamsters, a species quite susceptible to prion disease, has asparagines at residues 170 and 174 of its amino acid sequence, whereas rabbits, which are less susceptible to prion disease, incorporate serine at the equivalent positions. In the previously determined structure of wild-type rabbit PrP^C^ 121–230 [27], the sidechain of S174 forms a hydrogen bond with the backbone of N171 and *vice versa*, creating a hydrophobic staple helix cap motif [34]. We hypothesized that these sequence differences affect the formation of the helix-cap and consequently the folding behavior of PrP, its refolding to the β-state, and thereby its conversion to the infectious form.

To test this hypothesis, we introduced the single mutations S170N and S174N as well as the double mutation S170N/S174N into rabbit PrP^C^ 121–230, successively changing the rabbit sequence in the β2−α2 loop to that of wild-type hamster PrP^C^. Similarly, the single mutants N170S and N174S as well as the double mutant N170S/N174S of hamster PrP^C^ 121–231 were constructed, to stepwise transform its β2−α2 loop to the wild-type rabbit PrP^C^ sequence.

The structures of the S170N, S174N and S170N/S174N mutants of rabbit PrP^C^ 121–230 were solved by x-ray crystallography to resolutions of 1.4 Å, 1.6 Å and 1.5 Å, respectively. Unfortunately, exhaustive attempts to crystallize wild-type hamster PrP^C^ 121–231 and its three helix-cap mutants did not meet with success.

The structures of the three rabbit PrP^C^ 121–230 mutants show the same dimeric arrangement of PrP in the asymmetric unit that was observed in the wild-type structure published previously. Electron density was observed for residues 126–230 in one chain, whereas only residues 126–220 were visible in the other, most likely the result of differences in crystal packing. The dimer interface buries 1620 Å^2^ of surface area and involves 17 intermolecular hydrogen bonds and 6 salt bridges. Although a dimeric PrP has not been unequivocally identified under “native” solution conditions, the crystallographic arrangement is predicted to be stable by PISA [35]. The overall folds of the three rabbit PrP^C^ 121–230 mutants (S170N, S174N and S170N/S174N) are very similar to the wild-type structure. They all adopt the classic PrP^C^-fold with three α-helices encompassing residues 143–157 (helix-1), 171–193 (helix-2), and 199–230 (helix 3) as well as a small two-stranded, anti-parallel β-sheet consisting of residues 128–130 (β-strand 1) and 162–164 (β-strand 2). Comparing the wild-type rabbit PrP^C^ 121–230 and the S170N, S174N and S170N/S174N mutants, the r.m.s.d. between all four structures is 0.51Å for all backbone atoms.

**Figure 2:**
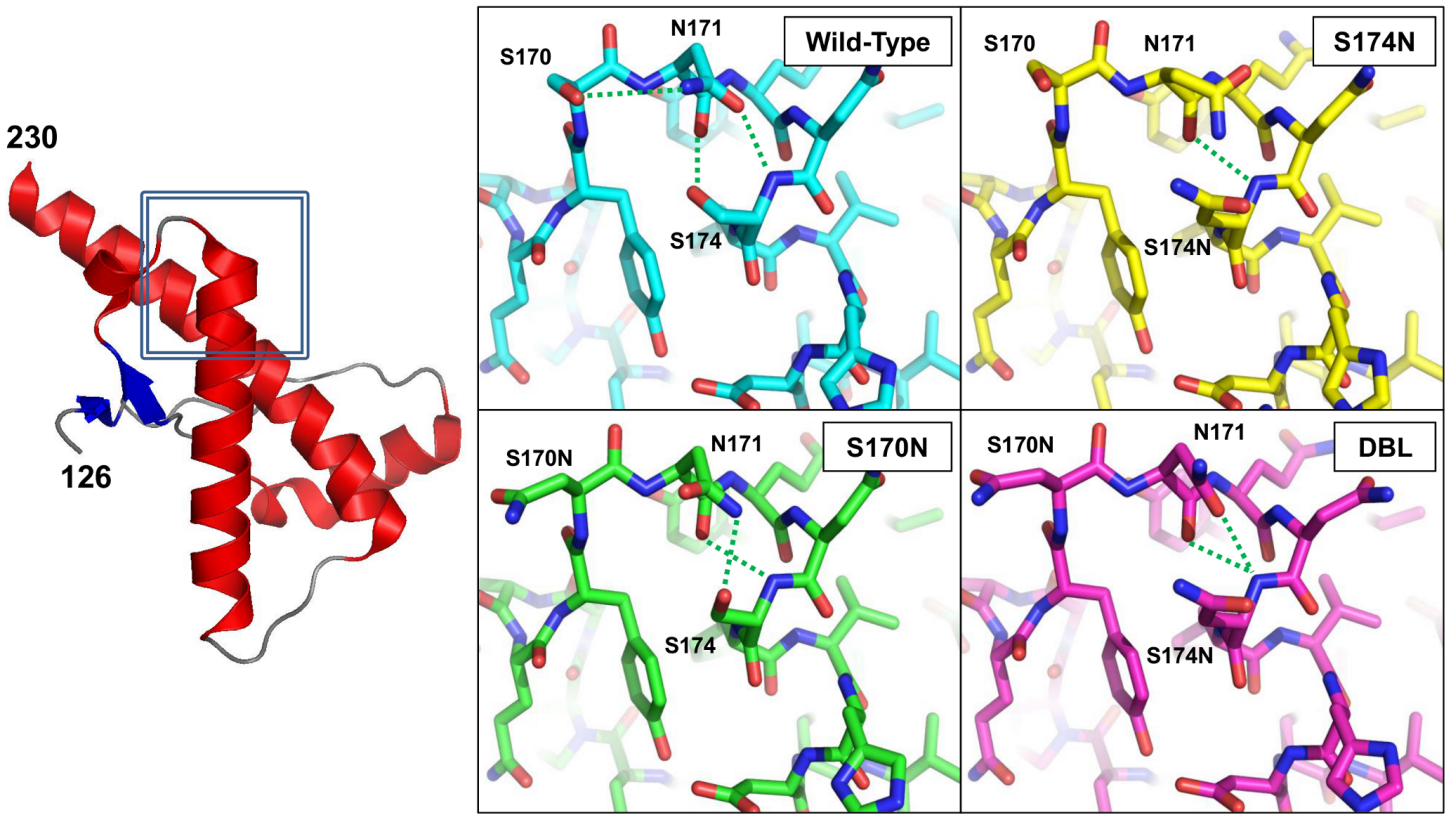
Representative structure of S170N, S174N and S170/S174N mutants of rabbit PrP^C^ 121–230. All three structures have the typical PrP fold of three α-helices and a small two-stranded β-sheet. All three structures displayed only 0.5 Å^2^ root-mean-squared deviation between equivalent Cα positions when compared to each other and to wild-type. Insets: close-up views of the residues forming the helix-cap in the wild-type and their equivalents in the three mutant structures of rabbit PrP^C^ 121–231. The reciprocal interactions between the backbone and side chains of S170 and S174 in the wild-type are ablated in the S174N and S170N/S174N mutant structures. The side chain of the mutant S170N is solvent exposed and disordered, but may weakly interact with the neighboring N171.

The β2−α2 loops in the three mutant structures consist of residues P165–N171 and are followed by the first turn of helix-2 formed by residues Q172–F175. Similar to the wild-type, residues N167–169 form a 3_10_-helical turn in all three mutants. In wild-type rabbit PrP, the side chain carbonyl oxygen of N171 hydrogen bonds to the backbone amide of S174 and the side chain hydroxyl of S174 interacts with the backbone carbonyl oxygen of N171. These reciprocal interactions, flanked by the hydrophobic interactions of Y169 and F175, form the basis for the hydrophobic staple helix-cap. This arrangement is altered in the S170N rabbit PrP^C^ structure with the side-chains of the mutant N170 and S174 no longer interacting with N171 to form the helix-cap. The electron density surrounding the N170 sidechain is fairly weak suggesting it may be disordered (Fig. 2 and Fig. S1B). The sidechain amide and backbone carbonyl of N171 can hydrogen bond with the sidechain hydroxyl oxygen and backbone amide of S174, respectively. In the crystal structure of the rabbit S174N mutant PrP^C^, the mutated N174 no longer hydrogen bonds with the backbone of N171, ablating the reciprocal interactions forming the helix cap and leaving the backbone of N171 exposed to solvent (Fig. 2 and Fig. S1C). The structure of the rabbit S170N/S174N double mutant PrP^C^ shows the features of both mutants with N170 remaining disordered as it is in the S170N mutant and N174 no longer hydrogen bonding to N171 (Fig. 2 and Fig. S1D), adding up to the same loss of helix cap-forming interactions found in the single S174N rabbit PrP^C^ mutant. Changing residues in the β2−α2 loop in rabbit PrP^C^ to those of hamster PrP^C^ causes key interactions in the helix-cap to be lost. Our inability to crystallize hamster PrP carrying mutations to the corresponding rabbit amino acids prevented us from testing whether a helix-cap could be introduced into hamster PrP^C^. However, we did seek to determine if these N170S, N174S, and N170S/N174 mutations would affect the stability and β-state refolding behavior of hamster PrP.

### Urea Induced Unfolding of Wild-type and Mutants of Rabbit and Hamster PrP^C^

**Figure 3:**
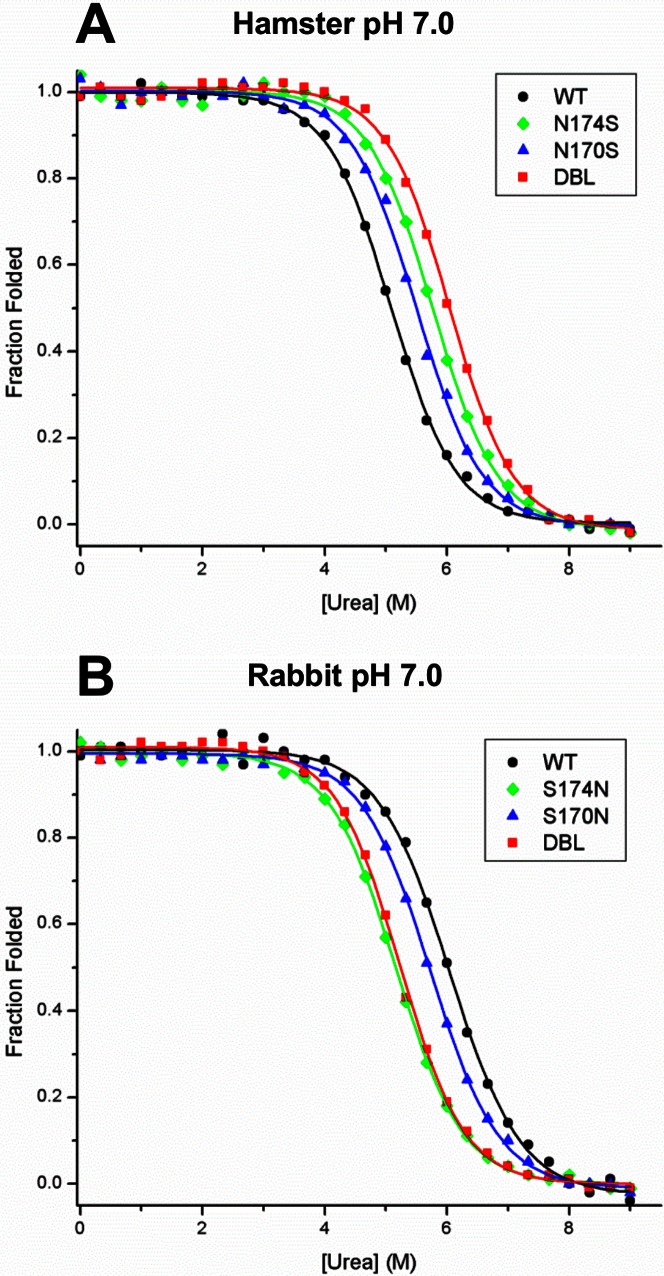
Urea-induced unfolding curves of wild-type and helix-cap mutants of rabbit and hamster PrP^C^ 121–230. Samples of wild-type and helix-cap mutants of hamster and rabbit PrP^C^ 121–230 were diluted to 10 µM in 50 mM sodium phosphate pH 7.0 with indicated concentrations of urea and incubated at room temperature for 72 hours. The proportion folded was then determined by measuring ellipticity by circular dichroism at 220 nm and normalizing between folded and unfolded baselines. A) In Hamster PrP^C^, the mutations of N170S and N174S caused a moderate increase in the free energy of unfolding of hamster PrP^C^ whereas the N170S/N174S double mutation showed an additive effect. B) In Rabbit PrP^C^, the mutation of S170N caused a small drop in the free energy of unfolding whereas for the mutations of S174N and S170N/S174N the decrease was more significant.

The presence of helix-caps at the N-termini of α-helices compensates for the decreased stability caused by exposure of backbone amide groups to solvent. Therefore, we hypothesized that the presence or absence of the helix-cap in rabbit and hamster PrP^C^ 121–231 would have a corresponding effect on their free energies of unfolding. We performed urea melts on the wild-type PrP^C^ 121–231 of rabbit and hamster as well as on the three helix-cap mutants of each of the two proteins (Fig. 3 **)**. We found that wild-type rabbit PrP^C^ 121–230 is significantly more stable than hamster PrP^C^ with free energies of unfolding of 6.51 and 5.6 kJ/mol, respectively (Table 2). The S170N single mutation reduced the rabbit PrP^C^ ΔG of unfolding to 5.9 kJ/mol, while the S174N change and the S170N/S174N double mutant displayed ΔGs of 5.45 and 5.7 kJ/mol, respectively (Fig. 3A). Conversely, asparagine to serine mutations in the hamster PrP^C^ sequence (Fig. 3B) increased the free energies of unfolding to 5.9 and 6.5 kJ/mol for mutants N170S and N174S, respectively. The N170S/N174S double mutant showed an additive effect with a ΔG of 6.8 kJ/mol (Table 2). These data support our hypothesis that introducing serine residues into positions 170 and 174 of hamster PrP^C^, residues that are involved in the helix-cap motif in rabbit PrP^C^, increase the protein’s stability; conversely, their replacement with asparagine residues in the rabbit PrP^C^ sequence will decrease the stability of this protein.

**Table 2:** Free energy of unfolding of wild-type rabbit and hamster PrP^C^ 121–231 as well as S170N, S174N, S170N/S174N mutants of rabbit PrP^C^ 121–230 and N170S, N174S, and N170S/N174S mutants of hamster PrP^C^.

Species/Mutation	ΔG_unfolding_	ΔΔG vs. WT	m	Urea ½
Hamster WT	5.68±0.12	–	−1.12±0.02	5.09±0.15
Hamster N170S	6.08±0.20	+0.4	−1.11±0.04	5.50±0.26
Hamster N174S	6.41±0.22	+0.7	−1.10±0.04	5.77±0.29
Hamster DBL	6.84±0.21	+1.1	−1.13±0.03	6.05±0.26
Rabbit WT	6.18±0.22	–	−1.02±0.04	6.04±0.31
Rabbit S170N	6.05±0.21	−0.2	−1.06±0.04	5.73±0.28
Rabbit S174N	5.60±0.13	−0.6	−1.08±0.02	5.16±0.17
Rabbit DBL	5.89±0.15	−0.3	−1.13±0.03	5.23±0.19

### β-State Propensity Measurements

Previous work in our lab has shown that species differences in the sequence of PrP can affect the propensity of PrP to populate the β-state upon incubation at low pH in the presence of urea. We decided to test whether the mutations we had made in the β2–α2 helix-cap also had these effects in rabbit and hamster PrP (121–231).

**Figure 4:**
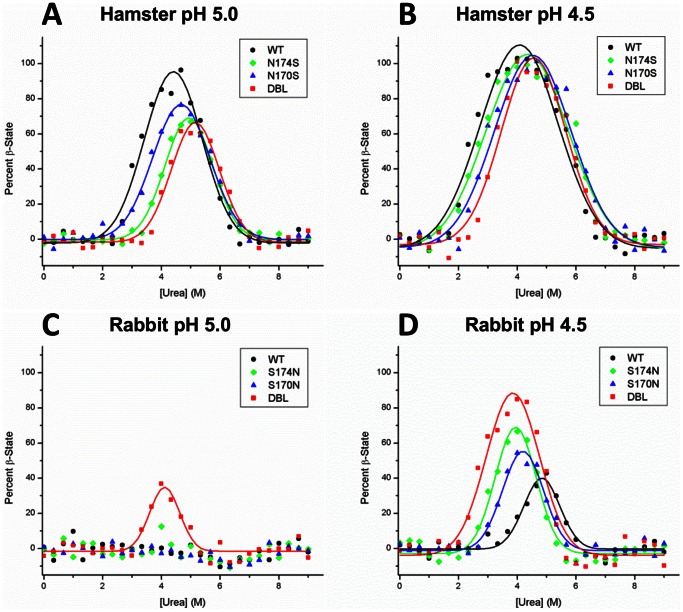
β-state propensity measurements of wild-type and helix-cap mutants of rabbit and hamster PrP^C^ 121–230. Samples were diluted to 10 µM in sodium acetate buffer at pH 5.0 or pH 4.5 with indicated concentrations of urea. After 72 hr incubation, the proportion of β-state was determined using the two-wavelength CD method (see Materials and Methods). (**A**) In hamster PrP at pH 5.0, the helix-cap mutations cause a decrease in the β-state population from 96% in the wild-type to 76%, 67%and 61% in the N170S, N174S and the N170S/N174S mutants, respectively. (**B**) At pH 4.5, the wild-type and helix-cap mutants all eventually populate the β-state to 100%, but to reach it they require increased urea concentrations. (**C**) In Rabbit PrP at pH 5.0, only the S174N and S170N/S174N mutants begin to populate the β-state to 15% and 35%, respectively. (**D**) At pH 4.5, the helix-cap mutations S170N, S174N and S170N/S174N cause an increase in the maximum β-state populations to 54%, 67%, and 85%, respectively, compared to 43% in the wild-type. Overall error of 4.5% was estimated from the difference between observed and fitted values.

We find that wild-type rabbit PrP (121–230) does not form the β-state at pH 5.0 (Fig. 4A) and forms a maximum of 42.8% β-state at pH 4.5 (Fig. 4B); compared to wild-type hamster PrP, which reaches 96% and 100% β-state at pH 5.0 and 4.5, respectively (Fig. 4C and D).

Introduction of the single mutations S170N and S174N and the double mutation S170N/S174N into rabbit PrP each cause respective increases in the β-state propensity of PrP. At pH 5.0, no β-state forms in the S170N mutant, whereas the S174N rabbit PrP begins to form the β-state to a maximum of 12.6% and the S170N/S174N double mutant forms a maximum of 37% (Fig. 4A). At pH 4.5, the S170N and S174N single mutants form 54% and 66.6% β-state and the double mutant sees a larger increase to 84.9% β-state (Fig. 4B).

Conversely, introduction of mutations into hamster PrP cause a decrease in the population of β-state PrP at low pH. While the wild type hamster PrP displays 100% β-state at pH 5, the single N170S and N174S mutations show a decrease to 76.4% and 66.9% β-state respectively, whereas the double mutant N170S/N174S reaches a maximum of 61.3% (Fig. 4C). At pH 4.5, all three hamster PrP mutants reach 100% β-state but there are slight differences in the urea concentration at which the β-state begins to form indicating differences in the stability of the PrP^C^ state of these mutants or their ability to form the β-state (Fig. 4D). These results demonstrate that single site mutations in this helix cap motif in PrP affect the β-state propensity PrP, and the mutations have an additive effect.

### Time-resolved Size-exclusion and Circular Dichroism

Previously we showed that the β-state of PrP consists of a mixture of β-state monomers and β-state octamers [27]. In order to determine whether species differences and mutations in PrP have an effect on the formation of β-state monomers and octamers, we monitored their proportions kinetically using circular dichroism and size-exclusion chromatography (SEC). Proteins at 100 µM concentration were dialyzed into their respective buffers at pH 4.0 without urea. At *t* = 0, they were diluted to 10 µM in identical buffer but with 4M urea and injected onto the columns at the indicated intervals.

**Figure 5:**
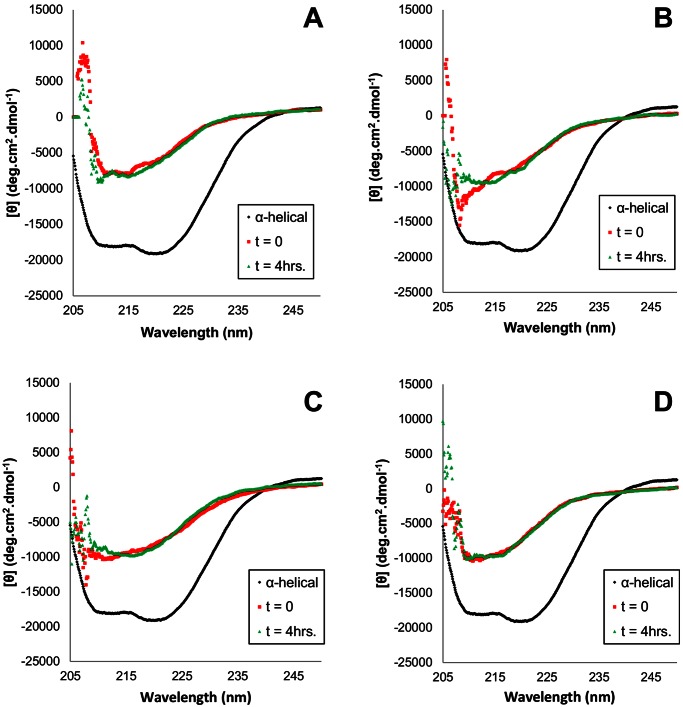
Circular dichroism wavelength scans of (A) wild-type hamster PrP 121–231, (C) hamster PrP 121–231 N170S/N174S, (B) wild-type rabbit PrP 121–230, (D) rabbit PrP 121–230 S170N/S174N. At *t* = 0, samples of 100 µM PrP in 50 mM sodium acetate pH 4.0, 80 mM NaCl were diluted to a final PrP concentration of 10 µM and 4 M urea in identical buffer. Circular dichroism wavelength scans were then performed at *t = *0 and *t* = 4 hrs between 205–250 nm at 0.1 nm intervals. The CD spectrum of α-helical PrP^C^ is included for comparison.

**Figure 6:**
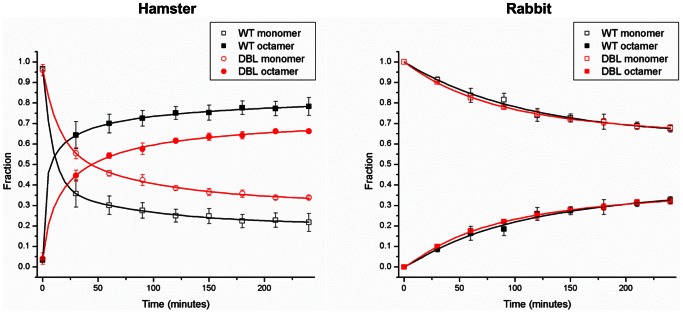
Time-resolved size exclusion chromatography analysis of wild-type and double helix-cap mutants of rabbit and hamster PrP. At *t* = 0, samples were diluted to a final PrP concentration of 10 µM in target buffer and immediately injected onto an S200 10/30 column. Fractional concentrations of monomer and octamer were calculated; no intermediate species were detected.

Upon diluting the concentrated stock into 4 M urea at pH 4.0, CD wavelength scans of both the hamster wild-type and N170S/N174S double mutant show them converting immediately to primarily β-sheet secondary structure (Fig. 5). SEC elution profiles of both show nearly 100% monomer at *t* = 0 which converts to a maximum 78% octamer in the wild-type and 65% octamer in the hamster double mutant after 4 hours; no intermediate species were detected (Fig. 6A). The extent of octamer assembly is slightly lower in the double mutant compared to the wild-type hamster PrP. The CD-signals for both the wild-type hamster and double mutant do not change significantly over the course of 4 hours, indicating constant β-structure (Fig. 5).

Similar to hamster PrP, both the wild-type and S170N/S174N rabbit PrP convert to primarily β-sheet secondary structure immediately after dilution to 10 µm PrP in 4 M urea (Fig. 5). However, the SEC elution profiles indicate that the assembly of β-state monomers into β-octamers is significantly slower in rabbit wild-type and double mutant compared to the hamster constructs (Fig. 6B). Both rabbit wild-type and double mutant at *t* = 0 remained monomeric, indicating that initially these both exist as β-state monomers. Over time the proportion of octamers in both the wild-type and double mutant increases, to a maximum of 33% after 4 hours; again no intermediate species were detected. In contrast to what had been seen with wild-type and mutant hamster PrP, rates and extent of β-octamer formation did not differ for wild-type and S170N/S174N rabbit PrP.

These results confirm our earlier observation that the β-state of PrP consists of mixtures of β-sheet-rich monomer and octamer. In addition, we observed that the extent of assembly of β-state monomers into octamers differs between species and that mutations in the sequence of PrP can influence these transformations.

## Discussion and Conclusions

### Comparison of Rabbit PrP 121–231 Crystal Structures to other PrP^C^ Structures

The crystal structures of the S170N, S174N, and S170N/S174N mutants of rabbit PrP^C^ (121–231) show that hydrogen bonding interactions observed in the helix-cap of wild-type rabbit PrP^C^ can be removed by mutating the serine residues involved. Few mammalian species have serine at position 174 of their PrP sequence; pigs are one of them and, interestingly, also display a low susceptibility to prion disease. Many susceptible species have asparagine at positions 170 and 174; by mutating the rabbit PrP^C^ serines to asparagines we have shown that interactions involving serine-174 in the rabbit PrP^C^ helix-cap are disrupted suggesting that this motif may be absent in PrP^C^ from species that are more susceptible to prion disease. In support of this idea, Vorberg *et al*. showed that the N173S mutation in mouse PrP (equivalent to rabbit residue 174) imparts resistance against the RML strain of scrapie prions in a neuroblastoma cell culture model [11]. However, the PrP sequence from elk, a species susceptible to chronic wasting disease, incorporates N170 and T174. Expression of the S170N/N174T mutant using a mouse model showed an increase in prion disease susceptibility [19]. This indicates that the presence or absence of S174 and its role in the helix cap may play a role in infectivity.

Structural differences within the β2–α2 loop of various PrP^C^ proteins had already been observed when their first structures were determined. Differences in the mobility involving the β2–α2 loop were described comparing the NMR structures of mouse and hamster PrP [13], [36], which contained mobile and rigid β2–α2 loops, respectively. Gossert *et al.* showed that the structure of elk PrP^C^ incorporated a rigid β2−α2 loop, which could be introduced into mouse PrP by mutating the mouse N173 to threonine (equivalent to 174 in other species) [16]. It was hypothesized that the differences in mobility could be caused by differences in hydrogen bonding within the β2−α2, loop but no specific bonds could be assigned. Subsequent structural work has suggested that the mobile or rigid loop may be a determinant in a species’ susceptibility to prion disease [17], [37]. A recent NMR structure of rabbit PrP^C^ showed differences in stability and hydrogen bonding throughout the molecule when Ser 174 is mutated to Asn, but no specific change in interactions involving the residue where detected [38].

Our structures and biophysical characterization of the wild-type and helix cap mutants of rabbit and hamster PrP have identified a structural motif that clearly affects the folding behavior of PrP. Other recent studies have found additional features of rabbit PrP that may also contribute to the reduced susceptibility of rabbits to prion disease. Amino acid differences present in the C-terminus of rabbit PrP have also been shown to interfere with PrP^Sc^ formation [39] and molecular dynamics simulations have suggested that salt bridges between D177-R163 and D201-R155 may increase global stability, preventing conversion to PrP^Sc^ [40], [41]. Further understanding of the relationship between these features and species susceptibility *in vivo* would provide insight into a biophysical mechanism of the convesion of PrP to the infectious form and the pathogenesis of prion disease.

### Effect of Mutations on β-state Propensity

The result of our urea denaturation experiments involving wild-type rabbit PrP and several of its mutants show that disruption of the helix-cap reduces the folding stability of PrP^C^. Conversely, introduction of serine residues involved in the rabbit PrP^C^ helix-cap into hamster PrP^C^ increases the latter’s stability. Although the helix-cap could not be directly observed in the hamster PrP mutants due to our inability to crystallize it, the observed increase in stability similar to that of the wild-type rabbit protein makes its presence probable. Interestingly, the scale of ΔΔG caused by the mutations (Table 2) is approximately the scale expected by the gain or loss of a hydrogen bond, giving further weight to the idea that this helix cap motif contributes to global stability. In addition, the presence of residues involved in the helix-cap also affects the propensity of rabbit and hamster PrP^C^ (121-230) to populate the β-state. Under conditions that promote β-state formation, amino acid changes in the S170N, S174N and S170N/S174N mutants of rabbit PrP^C^ cause successive increases in the population of the β-state at equilibrium, whereas the reverse mutations in hamster PrP (N170S, N174S and N170S/N174S) cause successive decreases in β-state population. This indicates that the helix-cap plays a role in preserving the α-helical fold of PrP^C^ and limits misfolding to the β-state. Without a detailed structure of the β-state it is difficult to speculate on how the helix-cap would affect its structure and formation. However, it has been shown that in the urea induced unfolding of PrP from various species, the β-sheet portion of PrP^C^ unfolds first, followed by helix-2 and 3 [42]. The helix-cap is present at the junction between these two potions of PrP^C^ and it may in some inhibit their dissociation and formation of the β-state.

Our data provide clues into how sequence differences affect the biophysical behavior of PrP. Determining the effects of mutations and species differences on prion infectivity has been hampered by the lack of detailed structural information regarding the infectious form, PrP^Sc^. To circumvent this, many groups have studied the misfolding of PrP *in vitro* using the recombinant β-state of PrP. To date, β-state PrP has not been shown to be infectious on its own although complex procedures have been published that achieve infectivity but require additional cofactors [43], [44]. So, it is still unclear how the β-state relates to the infectious form *in vivo*. However, several similarities between the β-state and the infectious form have been observed: The β-state is β-sheet rich and oligomeric [20], [21], [23], [24] as is the most infectious form of PrP^Sc^. It can also be protease resistant and toxic to cells *in vitro* [22], [25]. Previous work in our lab has shown that the propensity to refold into the β-state also correlates to prion disease susceptibility between species and we have now linked this finding to single amino acid differences in the sequence of PrP, similar to what was observed *in vivo* [9], [10]. Although the β-state probably does not represent an absolute replica of PrP^Sc^, the observed similarities argue that the β-state is well suited for study of PrP folding behavior *in vitro* providing a basis to decide which observations are the most promising candidates for testing in *in vivo* models.

### Kinetics of Formation of the β-State and the Transmission Barrier

Previous work has shown that the β-state of PrP proteins consists of β-sheet-rich monomers that assemble into octamers [27]. We also found that under β-state forming conditions both PrP^C^ 121–230 proteins from rabbit and hamster rapidly convert to the β-state monomeric form. However, the rate and extent of assembly of β-state monomers into octamers differs between species and is also affected by single amino acid changes in the helix-cap. This suggests that PrP from susceptible species assembles into octamers more quickly and to a greater extent than PrP from a resistant species, further supporting a connection between the β-state propensity and the susceptibility of a given species to prion disease.

Hypotheses regarding the transmission barrier have proposed that the rate of conversion of PrP^C^ to PrP^Sc^ plays an important role in prion disease pathogenesis. In a recent publication, Sandberg *et al.* report that prion disease pathogenesis occurs in two stages, an accumulation of prion titer to a plateau phase followed by the onset of clinical symptoms [45]. Our data now demonstrate that the PrP sequence from a given species can affect the rate and extent of misfolding of PrP to the β-state and its assembly into oligomers. This suggests that not only the level of expression but also the rate of conversion of PrP^C^ to PrP^Sc^ and the latter’s accumulation may also affect the pathogenesis of prion disease. PrP^C^ from less susceptible species may have sequence and structural features, such as the helix cap motif we have observed, that reduce the rate and extent to which conversion occurs, allowing the cell to compensate through proteolysis and clearance. While PrP^C^ from susceptible species, lacking such structural motifs, may convert rapidly and efficiently to PrP^Sc^, reaching concentrations that bring about clinical symptoms.

Additional factors must also be taken into consideration. A recent publication has demonstrated that although rabbits are less susceptible to infection with PrP^Sc^ from other species, they are still susceptible to prion disease albeit with a much lower attack rate and longer initial pre-symptomatic incubation periods [46]. Additionally, infectious material isolated from infected rabbits is able to re-infect other rabbits with a much higher attack rate and shorter incubation time in an example of prion adaptation. This demonstrates that although the sequence and structural features of PrP can lower prion disease susceptibility, they may not lead to complete immunity.

The effect of altered interactions within the helix-cap region on the conversion of PrP^C^ to the β-state suggests that this region plays an important role in the mechanism of conversion of PrP^C^ to PrP^Sc^. In the search for therapeutics and methods to prevent the conversion of PrP^C^ to PrP^Sc^ it should be beneficial to focus efforts on regions such as the β2−α2 loop that have been shown to affect susceptibility to disease transmission.

## Supporting Information

Figure S1 2F_O_-F_C_ electron density map at 1.5δ overlaid on the β2−α2 loops from respective structures of (A) wild-type rabbit PrP 121–230 (B) S170N mutant rabbit PrP 121–230, (C) S174N mutant rabbit PrP 121–230 and (D) S170N/S174N mutant rabbit.
(PDF)
