# Supplementary material for: N-Terminal Helix-Cap in α-Helix 2 Modulates β-State Misfolding in Rabbit and Hamster Prion Proteins
Source: PLoS One. 2013 May 10;8(5):e63047. doi: 10.1371/journal.pone.0063047 (PMC3651167; doi:10.1371/journal.pone.0063047)
Supplement: Figure S1 — 2FO-FC electron density map at 1.5δ overlaid on the β2−α2 loops from respective structures of (A) wild-type rabbit PrP 121–230 (B) S170N mutant rabbit PrP 121–230, (C) S174N mutant rabbit PrP 121–230 and (D) S170N/S174N mutant rabbit. (PDF) [file pone.0063047.s001.pdf]

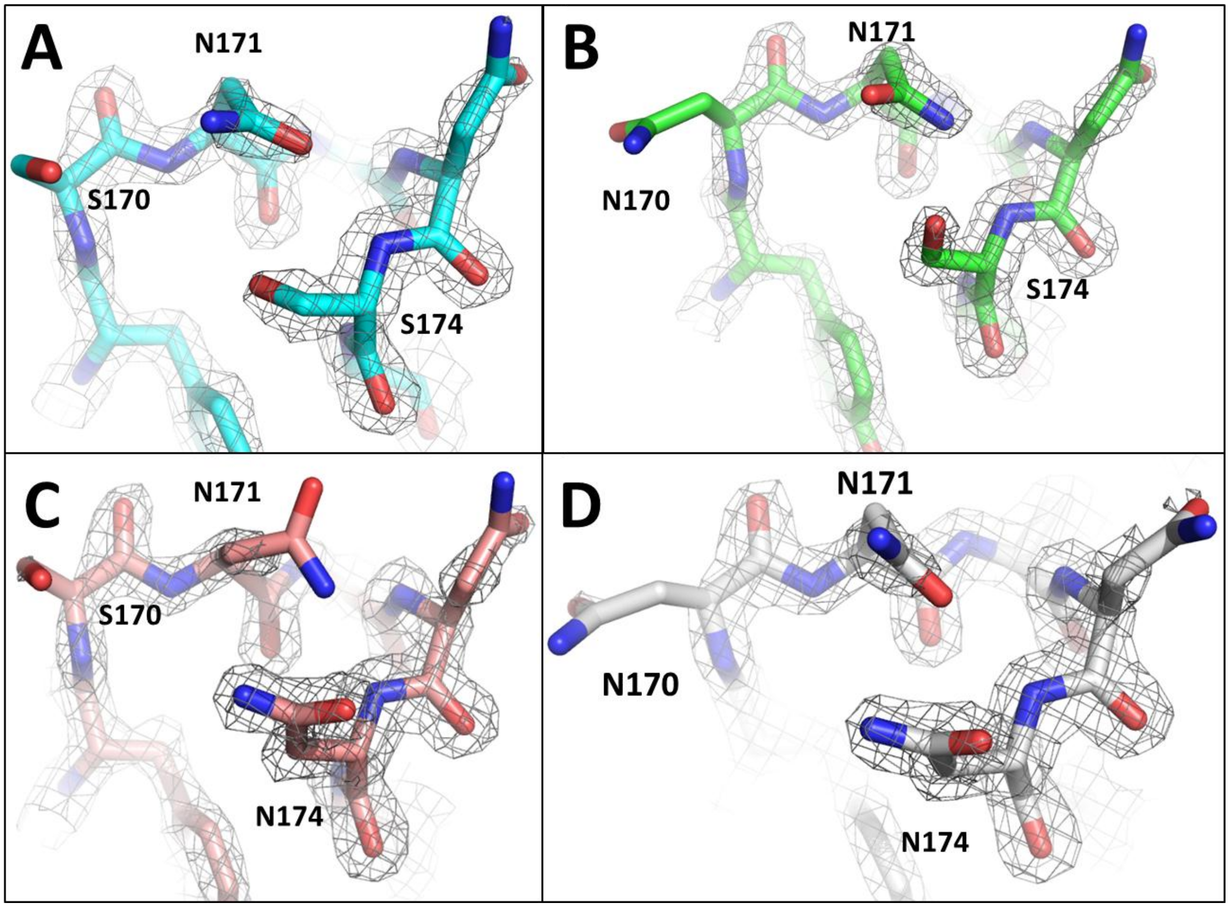

Figure S1: $2F_O$-F_C electron density map at 1.5δ overlaid on the β2-α2 loops from respective structures of (A) wild-type rabbit PrP^C 121-230 (B) S170N mutant rabbit PrP^C 121-230, (C) S174N mutant rabbit PrP^C 121-230 and (D) S170N/S174N mutant rabbit PrP^C 121-230.
